# Neighborhood-based physical activity differences: Evaluation of the effect of health promotion program

**DOI:** 10.1371/journal.pone.0192115

**Published:** 2018-02-05

**Authors:** Amanda Cristina de Souza Andrade, Sueli Aparecida Mingoti, Amanda Paula Fernandes, Roseli Gomes de Andrade, Amélia Augusta de Lima Friche, César Coelho Xavier, Fernando Augusto Proietti, Ana V. Diez-Roux, Waleska Teixeira Caiaffa

**Affiliations:** 1 Faculty of Medicine, Federal University of Minas Gerais, Belo Horizonte, Brazil; 2 Belo Horizonte Observatory for Urban Health, Belo Horizonte, Brazil; 3 Institute of Exact Sciences, Federal University of Minas Gerais, Belo Horizonte, Brazil; 4 Faculty of Health and Human Ecology, Vespasiano, Brazil; 5 School of Public Health, Drexel University, Philadelphia, Pennsylvania, United States of America; Indiana University, UNITED STATES

## Abstract

**Introduction:**

The practice of physical activity is an important factor in the prevention of health problems. However, a small portion of the population is physically active. Recent reviews show that physical activity classes in community settings have the potential to increase population levels of physical activity and reduce health inequalities.

**Objective:**

To evaluate the effect of the *Academias da Cidade* Program in Belo Horizonte on the practice of physical activity in leisure time (PALT) by non-users living near the program centers.

**Methods:**

We conducted a home-based health survey in Belo Horizonte (2008–2009) with 1,581 adults who were non-users of the program and who lived within a 1,500-meter radius of one active program center (exposed group) and two nonoperational centers with sites reserved for their construction (unexposed group). We collected data on PALT levels (≥150 minutes/week), which was measured with the Physical Activity International Questionnaire and analyzed with binary logistic regression using the Generalized Estimating Equations method. The propensity score was used as an adjustment variable to control the potential confusion in the measures of effect of exposure studied.

**Results:**

The overall prevalence of the PALT was 26.5% in the exposed group and 22.7% in the unexposed group. The exposed group was more likely to be active in leisure time (OR = 1.05; CI 95%: 1.01–1.10). When considering the interaction between exposed group and distance, individuals in the exposed group who lived less than 500 meters from the program center were more likely to be active in leisure time (OR = 1.18, CI 95%: 1.03–1.35) compared to their counterparts.

**Conclusion:**

Promoting physical activity in the community can favorably affect PALT levels among residents, especially those living closest to intervention centers. We believe the *Academias da Cidade* Program is a promising strategy to facilitate the access to appropriate spaces for the practice of physical activity and contribute to increase the levels physical activity within populations.

## Introduction

The promotion of physical activity is one of the top public health priority strategies as lack of physical exercise is one of the main risk factors for the development of chronic non-transmittable diseases, and frequently observed among populations. The adoption of an active lifestyle not only depends on individual behaviors and choices, but also on the interaction between individuals, environment and public policies[1].

The impact of the physical and social environment in health and health-related events has been increasingly studied[1]. A growing number of studies have shown that the environment plays an important role in promoting global physical activity or even in specific behaviors[2]. Access to places where exercise can be carried out is one of the most important factors in promoting higher levels of physical activity within the population[3–5].

In this context, community-based interventions have been designed as strategic actions for the mitigation of inequities, through the promotion of physical activity and changes in the environment that allow the access to appropriate spaces[6–8]. In Brazil, programs to promote physical activity include the *Academias da Cidade* Program, which was initially implemented in some Brazilian cities and, more recently, The Health Academy Program, implemented at the national level. Both are models of intervention for health promotion, aimed at encouraging and guiding the practice of physical activity in public spaces with appropriate infrastructures and qualified professionals[9].

The evaluation of these interventions is essential to determine their effectiveness on the health and access of the population, as well as the quality of the actions performed[9]. Two recent systematic reviews have highlighted the lack of studies focused on the assessment of physical activity interventions at the community level in Latin America[7, 8]. In Brazil, longitudinal data on these types of programs are lacking, and experiments designed in places where the programs have already been implemented and are ongoing for a long time are not feasible[6, 10]. The use of more robust analysis methods is necessary, including natural experiments and the use of the propensity score to evaluate the effectiveness of an intervention, even in the absence of randomization between the comparison groups[6, 11, 12].

Thus, the objective of this study was to evaluate the effect of the *Academias da Cidade* Program in Belo Horizonte, Brazil, on the practice of physical activity in leisure time of non-users living near the program centers. Non-users of the program correspond to individuals residing in the radius of up to 1,500 meters from the active program center (named exposed group) and to the residents in the same radius definition in two other neighborhoods planned for future construction (named the unexposed group). We hypothesized that the program, an intervention model for health promotion in the community, was able to increase the physical activity levels of the general population, and not only of its direct users.

## Materials and methods

### *Academias da Cidade* Program in Belo Horizonte

The *Academias da Cidade* Program in Belo Horizonte was created in 2005 as a community intervention by the Municipal Health Department of the city of Belo Horizonte and supported by the Brazilian Ministry of Health. The first center was inaugurated in *Leste* district of the city and, between 2007 and 2008, the program expanded, with the construction and planning of new centers, to other regional cities. Currently, the municipality has 75 program centers integrated into the Brazilian Unified Health System and composed of spaces with infrastructure, equipment and qualified human resources to guide bodily practices, physical activity and healthy lifestyles[13, 14].

The program aims to promote physical activity and improve the quality of life of the population by providing access to free activities such as gymnastics, dance, games, sports, fights, and walking, all of which are guided by a Physical Education professional. The activities are offered preferably for adults over 18 years, three times a week and one hour a day in up to two times per day (morning, afternoon or evening). Admission is by referral from the basic health unit or by spontaneous desire. The centers are strategically installed in areas of social vulnerability and in public places[13, 14].

### BH Health Study

To evaluate the effect of the *Academias da Cidade* Program on the practice of physical activity in the leisure time of the population assigned to the program units, a home-based survey named *BH Health Study* was applied between 2008–2009 by the Belo Horizonte Observatory for Urban Health of the Federal University of Minas Gerais. The survey collected data from two of the nine districts of Belo Horizonte, *Oeste* and *Barreiro*, which were selected due to their geographic proximity and internal heterogeneity regarding several demographic, socioeconomic and health indicators. The evaluation of the program was one of the main objectives of the study as a whole[15].

At the time of the survey, one program center had been active for six months and three were planned to be constructed. The center was inaugurated in October 2008 in a sports center built to respond to a collective demand from the residents, which was possible due to the Participatory Budget[14].

The sample of the study was obtained using a stratified sampling design and three-stage cluster: (a) census tract, selected with different probabilities and with a sample size proportional to the total of stratum census tracts; (b) domicile, selected with a simple random sample of households registered in the database of the Belo Horizonte City Hall; (c) an adult resident (aged 18 or more)[15]. The stratification factor, which allowed the proportional presence of all socioeconomic levels in the sample[16], was the Health Vulnerability Index, a multidimensional indicator resulting from the combination of social, demographic, economic and health indicators geocoded by the census tract. The final sample of the original study was composed of 4,048 adults (18 years or more) not enrolled in the program, referred to as “non-users” in this study.

To ensure the representativeness of residents in the vicinity of the *Academias da Cidade* Program, the probabilities of selecting each census tract were differentiated according to the distance to the centers. The two census tracts closest to the active and planned centers were included in the survey without randomization. Those located less than 500 meters and between 500 and 1,000 meters were, respectively, 8 and 4 times more likely to be selected compared to census tracts located at more than 1,000 meters from any program center.

The present study included a subsample of 1,581 non-users adults living in the 1,500 meter radius of the active program center and of two centers with locations reserved for their future construction. Residents around the fourth center, not implemented, were removed from the analysis because their original building site was changed. This geographic delimitation was adopted based on the information that most users of the active center resided within a radius of 1,500 meters[17].

Data from the survey were collected through face-to-face interviews conducted by trained interviewers, with a questionnaire containing household information, habits and behaviors, sociodemographic data, and social and health determinants.

### Outcome variable

Physical activity in leisure time was measured using the Physical Activity International Questionnaire and obtained by multiplying the frequency (days/week) and the average duration (minutes/day) of walking, and of mild, moderate and vigorous activities, the latter multiplied by two. Individuals with a physical activity score ≥150 minutes/week were considered active[18].

### Exposure variables

To measure the effect of the *Academias da Cidade* Program, two comparison groups were defined: (a) exposed group, corresponding to non-users residing in a radius of up to 1,500 meters from the active program center; (b) unexposed group, which included non-users residing in the same geographic area of two nonoperational centers whose sites were reserved for future construction.

For both groups, we measured the Euclidean distance in meters (0-500m; 501–1,000m; 1,001–1,500m) of the participants’ homes to the place where the program center was operational or the site was reserved for construction.

### Adjustment variables

The adjustment variables considered included gender (female, male), age (in years), years of schooling (0 to 8, 9 to 11 and more than 12 years), family income (<2, 2 to 5, ≥5 minimum wages), residence time (in years) and census tract *per capita* income categorized as low, medium and high tertiles. Income was obtained by the ratio between the total monthly nominal income of permanent private households and the total population of each census tract available in the 2010 census[19].

### Other variables

Two other variables were included. The perception of physical environment, measured by the following: “In your neighborhood, how would you rate the following public sports and recreational areas?”, measured on a five-item Likert scale (very good to very bad). Barrier to physical activity in leisure time among inactive individuals in the three months prior to the interview was measured by the following question: "Does not know the proper / safe place to exercise" (yes and no).

### Statistical analysis

To control for the lack of homogeneity between the comparison groups in terms of sociodemographic variables, we used the propensity score as an adjustment variable to reduce possible bias in the estimation of the intervention effect on the practice of physical activity in leisure time. The propensity score is defined as the conditional probability of an individual receiving treatment or, in the case of this study, being exposed to the intervention, given its observed covariates. It is represented by a single variable that simultaneously considers all potential covariates of confusion. Thus, individuals with the same score have the same distribution of observed covariates, regardless of their exposure condition[11, 12].

The propensity score was estimated using a binary logistic model with the maximum likelihood method. The dependent variable was the group (exposed and unexposed groups) and the independent variables were characteristics such as gender, age, residence length, years of schooling and family income.

The association between physical activity in leisure time and the variables “group” and “distance to the center” was estimated by the odds ratio and its respective 95% confidence interval (CI 95%) with binary logistic regression using the Generalized Estimating Equations method (GEE). GEE and an exchangeable correlation structure were used because the observations were grouped in one specific structure (i.e. individuals were nested within census tracts)[20]. The final models were adjusted for census tract *per capita* income and propensity score.

We used a significance level of 5%. All analyses were performed using the Stata software, version 12 (Stata Corp., College Station, USA). The manipulation of the geographic data was carried out using the MapInfo software, version 8.5 (MapInfo Corp. LP, New York, United States).

This study was approved by the Research Ethics Committee of the Faculty of Medicine of the Federal University of Minas Gerais (ETIC 253/06). All interviewees were informed about the aims of the research and signed the Informed Consent Form, agreeing in participating of the study.

## Results

The sample used in this study was composed of 1,581 individuals, of which 508 were in the exposed group (32.1%) and 1,073 in the unexposed group (67.9%). Of these, 60.4% were female, mean age 43.4 (± 16.5) and 15.7 (± 12.3) residence years. Most of the interviewees reported 0 to 8 years of study (51.0%), family income of 2 to 5 minimum wages (50.2%) and 48.4% resided in census tracts with *per capita* income classified in the mean position. (Table 1).

**Table 1:** Sociodemographic characteristics of the exposed and unexposed groups, The BH Health Study, Belo Horizonte, 2008–2009.

Variable	Total (n = 1,581)	Group	
		Exposed (n = 508)	Unexposed (n = 1,073)
Gender (%) *			
Female	955 (60.4)	324 (63.8)	631 (58.8)
Male	626 (39.6)	184 (36.2)	442 (41.2)
Age in years (mean ± SD)	43.4 (16.5)	43.8 (16.1)	43.4 (16.7)
Residence time in years (mean ± SD) *	15.7 (12.3)	14.5 (11.9)	16.3 (12.5)
Schooling years (%)			
0 to 8	806 (51.0)	251 (49.4)	555 (51.7)
9 to 11	589 (37.2)	189 (37.2)	400 (37.3)
More than 12	186 (11.8)	68 (13.4)	118 (11.0)
Family income (%) *			
<2 mw	467 (29.5)	133 (26.2)	334 (31.1)
2 to 5 mw	794 (50.2)	272 (53.5)	522 (48.7)
> 5 mw	320 (20.3)	102 (20.3)	217 (20.2)
Census tract *per capita* income (%) *			
Low	669 (43.2)	124 (24.4)	545 (52.4)
Average	749 (48.4)	368 (72.4)	381 (36.7)
High	129 (8.3)	16 (3.2)	113 (10.9)

mw–minimum wages: R$ 415,00; SD–standard deviation

* p<0,05.

**Fig 1:**
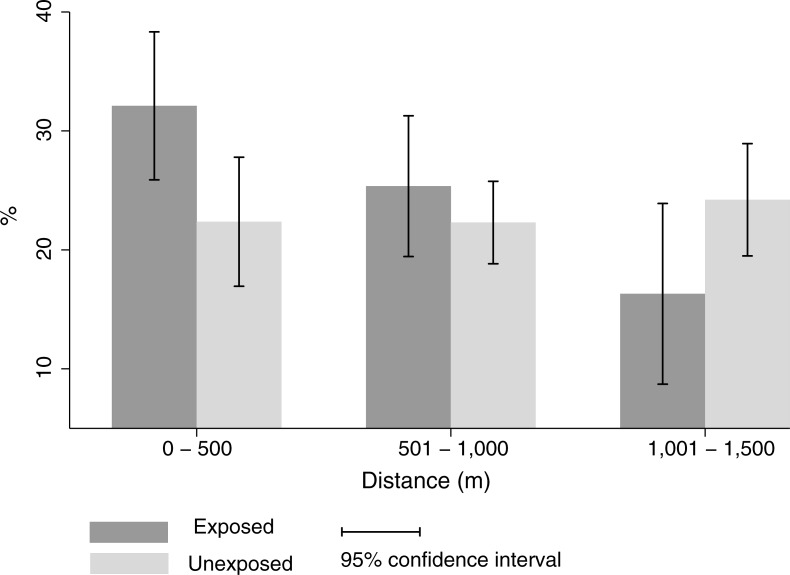
Proportion of active individuals in leisure time (≥150 minutes/week) in each group (exposed or unexposed groups) and distance to the program center. The BH Health Study, Belo Horizonte, 2008–2009.

The proportion of active individuals in leisure time (≥150 minutes/week) was 26.5% (CI 95%: 22.7–30.4) in the exposed group and 22.7% (CI 95%: 20.2–25.2) in the unexposed group. In the exposed group, we also detected a gradient between the proportion of active individuals and distance to the program center (32.1% for the 0-500m distance, 25.4% between 501 and 1,000m and 16.3% between 1,001 and 1,500m). This was not observed in the unexposed group (22.4% for the 0-500m distance, 22.3% between 501 and 1,000m and 24.2% between 1,001 and 1,500m) (Fig 1; S1 Table). The results observed in Fi 1 suggest that there is an interaction between the variables group and distance in relation to the practice of physical activity in leisure in this study.

**Fig 2:**
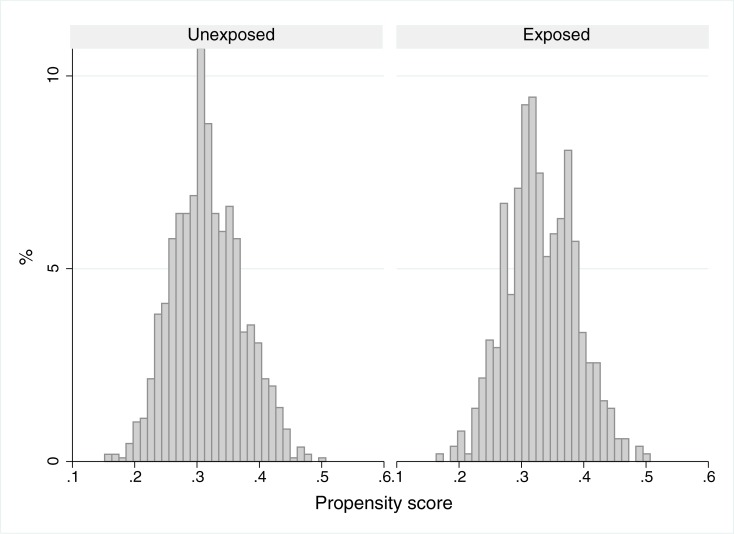
Distribution of the estimated propensity score for the exposed and unexposed groups. The BH Health Study, Belo Horizonte, 2008–2009.

We verified that the estimated probability of being in the exposed group was higher for women, individuals with family income between 2 and 5 minimum wages, older age and shorter residence time (Table 2). The variable schooling was not statistically significant. However, it was maintained in the model due to its importance as a marker of the socioeconomic level. Fig 2 shows the distribution of the estimated propensity score for the exposed and unexposed groups, and that the distribution was similar between the groups (p≥0,05; mean-comparison test). The propensity score allowed to balance the groups exposed and not exposed in relation as sociodemographic variables (S2 Table).

**Table 2:** Binary logistic model for the estimation of the propensity score. The BH Health Study, Belo Horizonte, 2008–2009.

Variable	OR	CI 95%	p
Gender (%)			
Female	1.26	1.02–1.58	0.039
Male	1.00		
Age in years	1.09	1.01–1.17	0.033
Residence time in years	0.98	0.97–0.99	0.001
Schooling years (%)			
0 to 8	1.00		
9 to 11	1.06	0.82–1.38	0.630
More than 12	1.33	0.90–1.96	0.143
Family income (%)			
<2 mw	1.00		
2 to 5 mw	1.36	1.05–1.76	0.018
> 5 mw	1.22	0.86–1.73	0.256

mw–minimum wages: R$ 415,00; OR–*odds ratio*; CI 95%: 95% confidence interval.

We then estimated the odds ratios and the confidence intervals of the association between physical activity in leisure time and the variables group and distance, adjusted by the income *per capita* of the census tract and propensity score (Table 3). In model 1, the exposed group revealed to be more likely to be active in leisure time (OR = 1.05, CI 95% 1.01–1.10). Including the variable “distance to the program center” in model 2 led to a loss of statistical significance of the variable “group”, which suggests an interaction between the two variables. In model 3, when considering the interaction between group and distance, a higher probability of being active in leisure time was observed for individuals in the exposed group and who lived less than 500 meters from the program center (OR = 1.18, CI 95%: 1.03–1.35).

**Table 3:** Estimates of the odds ratios and confidence intervals (95%) of the association between physical activity in leisure time and the variables “group” (exposed and unexposed groups) and “distance to the center” using a binary logistic model. The BH Health Study, Belo Horizonte, 2008–2009.

Variable	Model 1		Model 2		Model 3	
	OR (CI 95%)	p	OR (CI 95%)	p	OR (CI 95%)	p
Group						
Exposed	1.05 (1.01–1.10)	0.045	1.04 (0.99–1.09)	0.090	0.94 (0.85–1.04)	0.260
Unexposed	1.00		1.00		1.00	
Distance						
0–500m			1.03 (0.97–1.10)	0.330	0.97 (0.90–1.05)	0.522
501–1.000m			1.01 (0.95–1.06)	0.916	0.98 (0.92–1.04)	0.476
1.001–1.500m			1.00		1.00	
Group x Distance ^a^						
Intervention x 0–500m					**1.18 (1.03–1.35)**	**0.015**
Intervention x 501–1.000m					1.11 (0.98–1.26)	0.093
Intervention x 1.001–1.500m					1.00	

Models adjusted by the census tract *per capita* income and propensity score

ªinteraction between group and distance; OR–*odds ratio*; CI 95%: 95% confidence interval.

## Discussion

Community-based interventions to promote physical activity, such as the *Academias da Cidade* Program in Belo Horizonte, may favourably affect the practice of leisure physical activities among non-users living near the intervention centers. Individuals in the exposed group and who lived less than 500 meters from a program center were more likely to be active in leisure time than residents living at a higher distance.

These intervention models have been considered to be promising strategies to increase levels of physical activity of the population, as they promote environmental changes and community empowerment, and reduce inequalities in the access to physical activity[6–8]. Studies carried out in two Brazilian cities have demonstrated the potential of the *Academias da Cidade* Program in influencing the practice of physical activity among non-users, measured by the indirect effect of having seen or heard about the Program. These studies reported that people exposed to the Program, either visually or by participation, were more active than those who were not exposed[21–23].

A previous study carried out in the city of Belo Horizonte, with the same database used in the present study and with an exposed and unexposed group, was the first to show the effect of the Program on the practice of physical activity in leisure time among individuals residing at different distances to the centers[7]. Our results, in addition to corroborating the previous finding, also include the propensity score. This method allows controlling for the potential of confusion between intervention and outcome in observational studies, *i*.*e*. allows the comparison between the exposed and unexposed groups, such that the observed effect is attributed to the intervention and not to any differences between groups. The use of the propensity score is enough to remove the bias associated to the observed covariates used in its estimation[11, 12, 24].

The access to public spaces for the practice of physical activity is an important factor to reduce environmental barriers related to physical inactivity[3]. In the present study, the effect of the intervention was enhanced by a shorter distance to the program centers. People in the exposed group had a 5% higher odds of being active at leisure time than people in the unexposed group. Also, when considering the interaction between distance and exposure, individuals in the exposed group who lived less than 500 meters from a center were 18% more likely to be active in leisure time as compared to those in the unexposed group who lived less than 500 meters from a center. This result is in agreement with other studies reporting that a shorter distance to physical activity is associated with higher levels of physical activity[4, 5, 17, 22].

When analysing how individuals evaluated the public venues for sports and leisure available in their neighbourhood, we observed that 65.4% of those in the exposed group rated them as “very good” and “good”, while only 25.2% individuals in the unexposed group did the same. When considering the distance to the center, we observed a dose-response gradient evidenced by the effect of proximity to the program for the exposed group (88.7% for the 0-500m distance, 58.8% between 501 and 1000m and 25.3% between 1.001 and 1.500 m), but this was not observed in the unexposed group. In addition, those in the unexposed group who did not engage in leisure physical activity in the three months prior to the interview more frequently reported not knowing the place dedicated to sports and physical exercises (27.3% in the unexposed group versus 16.7% in the exposed group) (S3 Table). These results suggest that the Program, to some extent, modifies the way the environment is configured in terms of the availability of places for the practice of physical activity.

The adoption of an active lifestyle depends not only on the knowledge of the benefits associated with physical activity but also on the concrete conditions for its practice by different social groups[25]. The availability of services and public spaces varies according to the socioeconomic characteristics. Economically disadvantaged people are more likely to live in areas with more precarious or irregularly available services[26–28].

Thus, the prioritisation of community-based programs to encourage physical activity, such as the *Academias da Cidade* Program in Belo Horizonte, contributes to the reduction of inequities in the distribution of public equipment for physical activity for the general population, especially in areas of social vulnerability. In this context, environmental interventions and the formulation of public policies aimed at promoting physical activity, especially in urban areas, have great potential to increase the levels of physical activity of the general population.

Aspects related to the accessibility, aesthetics, maintenance and safety can be an important determinant for the use of public spaces for the practice of physical activity[5, 27]. In this sense, intersectoral actions are necessary to expand this intervention model and propose modifications to facilitate access to the equipment, such as more connected streets, the presence of sidewalks, greater illumination and security.

Some limitations of the current study have to be highlighted. For instance, information on the most variables analyzed in the current study was not available before and after the implementation of the intervention. However, the propensity score method was used as an adjustment variable, since its application is indicated when it is not possible to obtain data from the population before the intervention, or when the selection of the exposed and the unexposed groups was not randomized[29].

In the present study, the propensity score was used as the analysis strategy to ensure that the groups exposed and not unexposed to the intervention were comparable according to sociodemographic variables. The propensity score attempts to simulate randomization of individuals as occurs in randomized experimental studies. However, unlike randomization, the balancing achieved by propensity score is only on observed covariates rather than all possible confounders[11, 12].

The selection of only one active center and two with planned construction restricts the generalisation of the results obtained. On the other hand, it must be emphasised that the sample design of the basic survey sought to guarantee the representativeness of the affiliated population[15]. Also, the use of self-reported information, such as the practice of physical activity in leisure time, may be under- or overestimated in terms of duration and intensity of the activities performed by the individuals.

The Euclidean distance in meters between the homes of the interviewees and the program centers, one in operation and two with sites reserved for construction, was determined through the use of the Geographic Information System. The use of distance allowed the comparison of results between the groups with different levels of exposure to the intervention, increasing the internal validity of the study. Although this measure does not represent the actual displacement of individuals, because they do not consider the presence of natural or built obstacles and segment networks (streets and sidewalks), distance represents an objective measure of the environment and is not subjected to restrictions in self-reported data[30].

To better understand the effectiveness of physical activity interventions at the community level, it is recommended that the centers and their surrounding environment are evaluated using subjective methods and measurement objectives[30]. Future studies should include information before and after the implementation of the program, the control of effects not attributable to the intervention, and the use of natural experiments that allow making causal inferences about the effect of the intervention studied[31].

## Conclusions

Despite its limitations, the present study shows that the implementation of physical activity sites, such as the *Academias da Cidade* Program in Belo Horizonte, can increase the levels of physical activity of non-users of the program, and proximity to the centers of the program is a relevant factor to increase the effect of the intervention. The results of this study can serve as subsidies for actions of health promotion at the population level aimed at reducing the prevalence of inactive people, increasing levels of physical activity and coping with non-transmittable chronic diseases. This work also presents practical implications given the expansion of this intervention model at the federal level and its insertion in health promotion public policies.

## Supporting information

S1 Table Proportion of active individuals in leisure time (≥150 minutes/week) in each group (exposed or unexposed groups) and distance to the program center. The BH Health Study, Belo Horizonte, 2008–2009. CI 95%: 95% confidence interval.
(DOCX)

S2 Table Propensity score for the exposed and unexposed groups. The BH Health Study, Belo Horizonte, 2008–2009.
(DOCX)

S3 Table Perception of public area for sports and leisure available in neighbourhood of the exposed and unexposed groups, The BH Health Study, Belo Horizonte, 2008–2009. ^1^ 84 missing; ^2^ Did not engage in leisure physical activity in the three months prior to the interview.
(DOCX)
