# Supplementary material for: Neighborhood-based physical activity differences: Evaluation of the effect of health promotion program
Source: PLoS One. 2018 Feb 5;13(2):e0192115. doi: 10.1371/journal.pone.0192115 (PMC5798787; doi:10.1371/journal.pone.0192115)
Supplement: S1 Table — The BH Health Study, Belo Horizonte, 2008–2009. CI 95%: 95% confidence interval. (DOCX) [file pone.0192115.s001.docx]

S1 Table. Proportion of active individuals in leisure time (≥150 minutes/week) in each group (exposed or unexposed groups) and distance to the program center. The BH Health Study, Belo Horizonte, 2008-2009.

| Variable | Exposed Group | |  | Unexposed Group | |
| --- | --- | --- | --- | --- | --- |
|  | % | CI 95% |  | % | CI 95% |
| Distance |  |  |  |  |  |
| 0-500m | 32.1 | 25.9 - 38.3 |  | 22.4 | 16.9 - 27.8 |
| 501-1,000m | 25.4 | 19.4 - 31.3 |  | 22.3 | 18.8 - 25.8 |
| 1,001-1,500m | 16.3 | 8.7 - 23.9 |  | 24.2 | 19.5 - 28.9 |
| Total | 26.5 | 22.7 - 30.4 |  | 22.7 | 20.2 - 25.2 |

CI 95%: 95% confidence interval.
