# Supplementary material for: Neighborhood-based physical activity differences: Evaluation of the effect of health promotion program
Source: PLoS One. 2018 Feb 5;13(2):e0192115. doi: 10.1371/journal.pone.0192115 (PMC5798787; doi:10.1371/journal.pone.0192115)
Supplement: S2 Table — The BH Health Study, Belo Horizonte, 2008–2009. (DOCX) [file pone.0192115.s002.docx]

S2 Table. Propensity score for the exposed and unexposed groups. The BH Health Study, Belo Horizonte, 2008-2009.

| Group | n | Mean | Standard deviation | Minimum | Maximum | Percentile 25 | Percentile 50 | Percentile 75 |
| --- | --- | --- | --- | --- | --- | --- | --- | --- |
| Exposed | 508 | 0.33 | 0.06 | 0.17 | 0.51 | 0.29 | 0.33 | 0.37 |
| Unexposed | 1,073 | 0.32 | 0.06 | 0.15 | 0.51 | 0.28 | 0.31 | 0.35 |
| Total | 1,581 | 0.32 | 0.06 | 0.15 | 0.51 | 0.28 | 0.32 | 0.36 |
