# Supplementary material for: Neighborhood-based physical activity differences: Evaluation of the effect of health promotion program
Source: PLoS One. 2018 Feb 5;13(2):e0192115. doi: 10.1371/journal.pone.0192115 (PMC5798787; doi:10.1371/journal.pone.0192115)
Supplement: S3 Table — 1 84 missing; 2 Did not engage in leisure physical activity in the three months prior to the interview. (DOCX) [file pone.0192115.s003.docx]

S3 Table. Perception of public area for sports and leisure available in neighbourhood of the exposed and unexposed groups, The BH Health Study, Belo Horizonte, 2008-2009.

| Variable | Total | Exposed Group | | | |  | Unexposed Group | | | |
| --- | --- | --- | --- | --- | --- | --- | --- | --- | --- | --- |
|  |  | 0-500m | 501-1,000m | 1,001-1,500m | Total |  | 0-500m | 501-1,000m | 1,001-1,500m | Total |
| In your neighborhood, how would you rate the following public sports and recreational areas? ¹ | (n=1,497) | (n=204) | (n=194) | (n=87) | (n=485) |  | (n=208) | (n=510) | (n=294) | (n=1,012) |
| Very good | 29 (1.9) | 19 (9.3) | 5 (2.6) | 0 (0.0) | 24 (5.0) |  | 1 (0.5) | 0 (0.0) | 4 (1.4) | 5 (0.5) |
| Good | 543 (36.3) | 162 (79.4) | 109 (56.2) | 22 (25.3) | 293 (60.4) |  | 61 (29.3) | 113 (22.2) | 76 (25.8) | 250 (24.7) |
| Bad | 680 (45.4) | 20 (9.8) | 67 (34.5) | 48 (55.2) | 135 (27.8) |  | 105 (50.5) | 292 (57.2) | 148 (50.3) | 545 (53.8) |
| Very bad | 245 (16.4) | 3 (1.5) | 13 (6.70) | 17 (19.5) | 33 (6.8) |  | 41 (19.7) | 105 (20.6) | 66 (22.5) | 212 (21.0) |
|  |  |  |  |  |  |  |  |  |  |  |
| Does not know the proper / safe place to exercise ² | (n=1,029) | (n=128) | (n=126) | (n=64) | (n=318) |  | (n=148) | (n=362) | (n=201) | (n=711) |
| Yes | 247 (24.0) | 17 (13.3) | 22 (17.5) | 14 (21.9) | 53 (16.7) |  | 32 (21.6) | 103 (28.4) | 59 (29.3) | 194 (27.3) |
| No | 782 (76.0) | 111 (86.7) | 104 (82.5) | 50 (78.1) | 265 (83.3) |  | 116 (78.3) | 259 (71.5) | 142 (70.6) | 517 (72.7) |

¹ 84 missing; ² Did not engage in leisure physical activity in the three months prior to the interview.
